# The Perspectives of Patients and Health Professionals Regarding the Tuberculosis Control Programme in Recife, Brazil: A Contribution to Evaluation

**DOI:** 10.3390/pharmacy7020070

**Published:** 2019-06-19

**Authors:** Simone Santos Bezerra, Mara Pereira Guerreiro, Nathany França Pessoa, Maria Gabriela Pereira da Silva, Mirela Galvão de Barros, João José Ferreira Gomes, Maria Paula Athayde, Rodrigo Zeymer Auad, José Lamartine Soares Sobrinho

**Affiliations:** 1 Pharmaceutical Science Department, Federal University of Pernambuco-Recife, Pernambuco 50740-52, Brazil; t-hany18@hotmail.com (N.F.P.); maria_gabrielapsa@hotmail.com (M.G.P.d.S.); mirelagalvao@gmail.com (M.G.d.B.); joselamartinesoares@gmail.com (J.L.S.S.); 2 Escola Superior de Enfermagem de Lisboa, Unidade de Investigação e Desenvolvimento em Enfermagem (UI&DE), 1990-096 Lisboa, Portugal; mara.guerreiro@sapo.pt; 3 Portugal & Centro de Investigação Interdisciplinar Egas Moniz (CiiEM), 2829-511 Monte de Caparica, Portugal; 4 Faculty of Sciences, Universidade de Lisboa, 1749-016 Lisboa, Portugal; jjgomes@campus.ul.pt; 5 Otávio de Freitas Hospital-Recife, Pernambuco 50920-460, Brazil; paulathayde@uol.com.br; 6 State Technical School Professor Agamenon Magalhães, Recife 52050-385, Pernambuco, Brazil; rodzeymer@yahoo.com.br

**Keywords:** tuberculosis, program and health projects evaluation, patients’ perspectives, professionals’ perspectives

## Abstract

The study objective is to describe patients and professionals’ perspectives on the Tuberculosis Control Program (PCT) in Recife, Brazil, contributing to the program evaluation. A cross-sectional study was conducted in three purposively selected sites, representing the three levels of care in the public health system. All eligible PCT patients in sites A, B and C were invited to participate (n = 123). Physicians, nurses, pharmacists and community health agents providing care to PCT patients in these sites, plus their managers, were purposively selected. Data were collected by means of interviews with 44 patients and a questionnaire to 24 professionals. Instruments encompassed previously published items to capture stakeholders’ perspectives (16 and 12 closed-questions, respectively), grouped into categories. The overall evaluation by patients was unsatisfactory (median score 35%; third quartile below 50%; interquartile range 21.9%). Analysis of scores by categories showed that opinions about organizational accessibility were significantly worse than about economic and geographical accessibility, taken together. Overall the median score attributed by professionals was 52% (third quartile below 65%). Professionals had significantly worse opinions about diagnosis, clinical and laboratory assistance. Patients and professionals’ perspectives highlight potential opportunities for improvement. Our findings can be used by managers as a starting point for shared decision-making, potentially contributing to a better performance of the PCT in Recife and, consequently, reducing the risk posed by tuberculosis.

## 1. Introduction

Tuberculosis (TB) is currently the second leading cause of death from infectious diseases worldwide, after human immunodeficiency virus (HIV) [1]. It remains a major health problem, especially in underdeveloped and developing countries [2]. In Brazil, disease control actions are part of the National Tuberculosis Control Program (PNCT), which is developed in the public network of the health system (Sistema Único de Saúde—SUS). The program intends to provide universal coverage for diagnosis and treatment of cases [3]. In 2001, TB management became decentralized and the responsibility of PNCT actions was attributed to primary health care. These actions can be implemented in these sites, via Basic Health Units (UBS) or by referring persons with TB to outpatient units; therefore, health professionals at different care levels are involved. Public health units staff includes pharmacists, who have direct patient contact; part of their role is dispensing TB medication.

A health program has been defined as “an organized response to reduce or eliminate a problem that, when it reaches its goal, improves the health of the population” [4]. It is widely accepted that evaluation is an integral part of health programs. In Brazil, there has been an increasing effort to produce evidence informing the reorganization of services provision, which has generated discussions about the evaluation of health programs at a governmental level and in the scientific community [5]. There are different approaches described in the literature with respect to evaluation in health [6]. A commonly cited one is based on the seminal work of Donabedian for assessing the quality of care, resorting to structure, process and outcome indicators [7]. A common aspect in different approaches is the importance of users and providers ’perspectives [8].

TB management requires integration of care among different levels, which poses additional challenges in respect to evaluation, especially in Brazil, due to the diversity of contexts of the health system in different regions [9]. Several studies have been published on the evaluation of the Tuberculosis Control Program in the northeast, north, southeast and other regions of Brazil [5,9,10,11,12,13]. These studies reported difficulties associated with unmet PCT goals and lack of successful integration of this program in primary health care; the relationship between TB and social vulnerability was again pointed out. Service performance in TB treatment faces two main obstacles: difficulties in accessing health services and actions plus lack of integration between the health care services network [5,9,10,11,12,13]. Challenges remain in controlling TB, in spite of the allocation of a USD 67 million budget for the Brazilian strategic plan against tuberculosis in 2017 [14].

We are unaware of studies that simultaneously present the perspectives of patients and health professionals about the PCT in the Northeast of Brazil. It is to this area that the present study turns. By describing the perspectives of patients and professionals about the PCT in Recife, this study intends to contribute to its evaluation.

## 2. Materials and Methods

### 2.1. Setting

The research was conducted in Recife, the capital of the State of Pernambuco, between July 2014 and February 2015. Recife is divided into eight Political-Administrative Regions (or Sanitary Districts). The sites were purposively selected to represent the three levels of SUS complexity in three sanitary districts (I, II and V). Five health units were included: two Basic Health Units and one polyclinic in Sanitary District II (site A); a university hospital of medium complexity (site B) and a hospital of high complexity (site C).

### 2.2. Population and Sampling

The population was comprised by outpatient patients aged 16 years and older receiving TB treatment in sites A, B and C. We considered physicians, nurses, pharmacists and community health agents providing care to PCT patients in these sites as professionals, as well as their managers.

All eligible PCT patients in sites A, B and C were invited to participate (n = 123). In total, 44 patients were interviewed: 28 patients from site C, eight from site A and eight from site B, yielding a response rate of 36%. Professionals were sampled purposively; all 24 professionals selected responded to the questionnaire (seven at site A, 11 at site B and six at site C).

### 2.3. Data Collection

#### 2.3.1. Interviews with Patients

The survey was administered by interviewers trained for this purpose. It included questions on socioeconomic variables (gender, age, family income, smoking status).

Perspectives about the PCT were collected by means of 16 closed questions, previously used by other researchers [9,15]. These evaluation criteria were grouped into the three categories, based on previous work [16]: Organizational, Economical and Geographical Accessibility. Answers were collected on a five-point scale previously used by the authors, ranging from 1 to 5 (never, almost never, sometimes, almost always, always, respectively). The criteria used in the evaluation and data obtained are presented in the “Results” section.

#### 2.3.2. Questionnaires for Professionals

The survey included questions on demographic variables (age, gender, profession and work place). Perspectives about the PCT were collected using 12 closed-questions adapted from Oliveira et al [17], grouped into four categories (q1–q4): Coverage and Reception; Diagnosis, Clinical and Laboratory Assistance; Treatment and Pharmaceutical Assistance; Integrality of care. Professionals were asked to score each question from 1 to 5, with the option “not applicable”.

### 2.4. Data Management and Analysis

The data obtained in paper surveys were manually entered into a Microsoft Excel spreadsheet; accuracy of responses in this file was double-checked. Missing values (1.5%) were excluded from the analysis.

A total score was calculated for each row, corresponding to a patient or a professional, by summing ratings of closed questions (Table 1 and Table 2) and dividing it by the number of applicable questions (i.e. number of questions answered by the respondent); the figure was then converted into a percentage. Statistical analysis was performed using R software. In addition to the descriptive statistics, Kruskal-Wallis or Wilcoxon tests were used to ascertain differences between variables of interest, depending on the nature of data and assumptions of each statistic test. A significance level of 0.05 was adopted in all tests.

### 2.5. Ethical Considerations

The study protocol was approved by the Research Ethics Committee of the first author’s institution, under the number 1413715. Ethical tenets of the Resolution 466/2012 of the National Health Council were followed [18]. All participants signed an informed consent term.

## 3. Results

### 3.1. Interviews with Patients

The majority of the patients were male (77%, 34/44); age ranged between 23 and 65 years (mean 45.4). Most had scarce economic resources, with a family income of less than four minimum wages or no income (97%, 43/44). Nine out of ten patients reported being non-smokers (40/44). About 40% of the patients had never been hospitalized due to TB (17/44), while approximately 40% had two or more TB-related hospitalizations (16/44).

**Figure 1:**
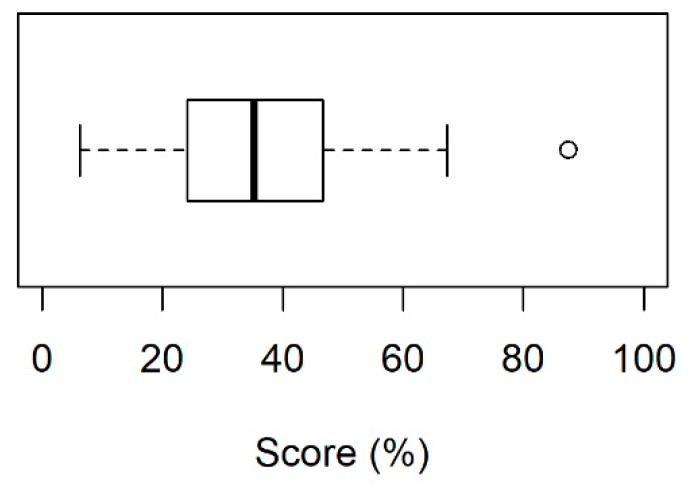
Patients’ overall opinions on the Tuberculosis Control Program in Recife-PE, Brazil.

Overall patients’ perspectives regarding the PCT were unfavorable; the median score was 35% (minimum 6%, maximum 88%) and the third quartile was below 50% (interquartile range 21.9%)-Figure 1.

There were differences between the median scores in the three sites (A = 27%, B = 37% and C = 36%), but these were not statistically significant (Kruskal-Wallis test; p = 0.3925).

The less positive scores attributed by patients (Table 1) included “home visits by health professionals related to TB treatment” (mean score 2.0, SD = 1.5) and “contact with advertising/campaign /educational work by health service professionals” (mean score 1.6, SD = 1.3)

**Figure 2:**
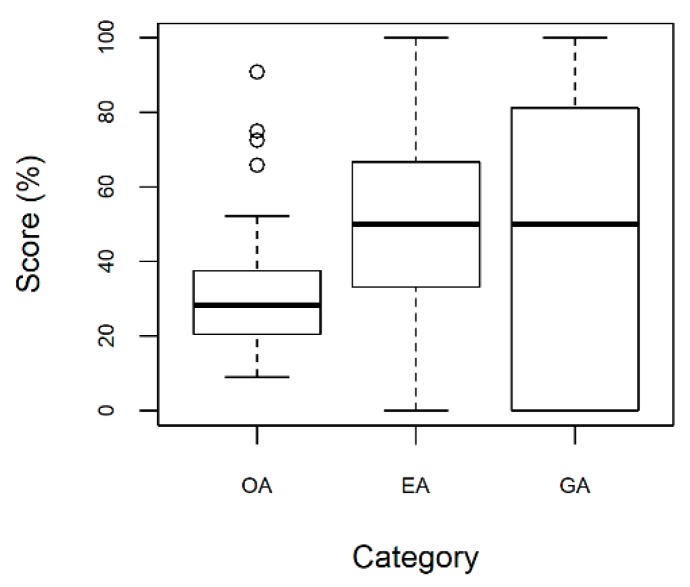
Patients’ opinion on the PCT by category, Recife-PE, Brazil.

Analysis of scores by categories (Figure 2) showed that opinions about organizational accessibility (OA) are significantly worse than opinions about economic accessibility (EA) and geographical accessibility, taken together (signed rank test, V = 715.5, p-value = 0.005; Paired t-test, t = 2.9196, p-value = 0.003). Organizational accessibility received the least favorable score (median 42.7% versus 60.0% and 60.0%, respectively).

### 3.2. Questionnaires for Professionals

Respondents were mostly women (87.5%, 21/24); age ranged between 30 and 55 years (mean 41.8). Nurses and physician comprised more than half of the respondents (33%, 8/24 and 25%, 6/24, respectively); followed by pharmacists (n = 5), managers (n = 3) and community health agents (n = 2).

Overall, professionals’ perspectives regarding the PCT were also unfavorable: the median score was 52% (minimum 22.9%, maximum 77.3%); the third quartile was below 65% (interquartile range 13.75). Table 2 presents professionals opinion on the PCT by item.

**Figure 3:**
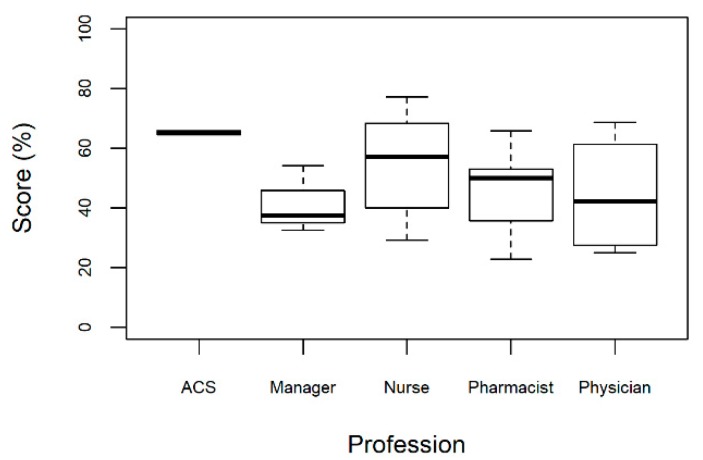
Opinions by professional group, Recife-PE, Brazil.

There were differences in the median scores by professional group (Figure 3). Community health agents (ACS) had a more positive perspective about the program (median score 65%) whilst managers and physicians offered negative perspectives (median score 38 and 42%, respectively). However, these differences were not statistically significant (Kruskal-Wallis test; p = 0.40).

Considering the three data collection sites, there were statistically significant differences regarding professional opinions on the PCT. The professionals working in site C presented the less favorable opinion in comparison to sites A and B, taken together (Wilcoxson test; p = 0.025).

**Figure 4:**
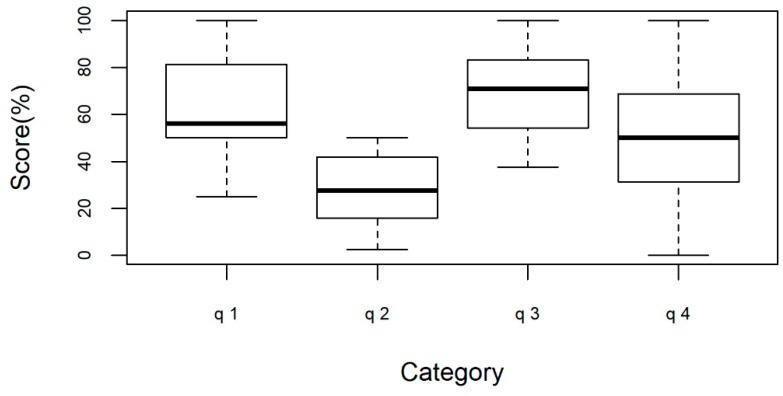
Professionals’ opinion on the PCT by category, Recife-PE, Brazil. (q1: Coverage and Reception; q2: Diagnosis, Clinical and Laboratory Assistance; q3: Treatment and Pharmaceutical Assistance; q4: Integrality of care).

Analysis of the median scores by category, depicted in Figure 4, showed that professionals had significantly worse opinions about “Diagnosis, Clinical and Laboratory Assistance” when compared with the other categories, taken together (Wilcoxson test, p < 0.01). Overall, the most favorable opinion held by professionals was about “Treatment and Pharmaceutical assistance”, followed by: “Coverage and Reception” and “Integrality of Care” (Figure 4).

## 4. Discussion

This study aimed to describe the perspectives of patients and professionals about the PCT in Recife, hence contributing to the evaluation of this program. Overall, patient and professional perspectives were unfavorable. The former scored aspects pertaining to organizational accessibility more unfavorably, while the latter expressed fewer positive opinions about diagnosis, clinical and laboratory assistance.

Items with low scores deserving discussion include “home visits by health professionals related to TB treatment” and “receiving information on TB and its treatment”, as they suggest an inability of the program to promote medication adherence and to tackle non-adherence problems or potential treatment discontinuation at a community level.

In Brazil, as in other countries, it has been shown that factors hindering access to treatment can lead to treatment discontinuation, which is associated with an increase in the cost of following-up patients and a rise in disease relapse [19,20]. With respect to geographical accessibility, dependence on motorized transportation for medical consultation did not emerge as problematic in our findings, contrasting with the findings of Arakawa et al [16]. The fact that organizational accessibility had the worst evaluation in the sites surveyed in Recife entails an opportunity. It might be easier for managers of health units to change aspects intrinsic to the organization, such as waiting time for medical consultations and availability of home visits, than extrinsic aspects, such as the need to use motorized transport to attend medical consultation. For example, receiving information on TB and its treatment, which obtained a low score in this research, may potentially be changed without additional resources. One possible approach is ensuring that pharmacists counsel patients in a structured and systematic fashion when dispensing TB medication. It should be borne in mind that insufficient information about the disease and its treatment can result in poor adherence and discontinuation of drug-therapy [21,22,23], which, in turn, contributes to an increase in drug resistance [24] and mortality [25]. Database research showed discontinuation rates urging attention, particularly in site B, as it exceeded the WHO 5% target [26,27]. Therefore, pharmacists delivering care to TB patients may be ideally positioned to implement more intensive interventions. The literature offers examples of how pharmacists can effectively contribute to reduce the burden of non-adherence in TB patients [28]. Pharmacists could also use dispensing data to signal non-adherent patients and tailor interventions addressing its causes.

Other aspects of organizational acceptability, such as providing information about TB and its treatment to people living with patients, and providing information on other health topics, may also be improved by resorting to pharmacists dispensing TB medication. 

No significant differences were found between the overall views of the various professional groups on the program. This may be due to the true absence of differences or to a sample size that is insufficient to reflect differences. Management of TB patients requires a multi-disciplinary approach; different professionals may offer valuable supplementary views. Pharmacists are part of the multi-disciplinary team, performing roles such ensuring a consistent supply of anti-TB drugs, promoting its rational use and helping to provide information to patients [29]. In this study pharmacists had a more favourable opinion about the PCT than physicians, but less favourable than nurses.

The less positive score attributed by professionals to the category “Diagnosis, Clinical and Laboratory Assistance” corroborates known difficulties, such as shortage of qualified human resources, the centralized and fragmented organization of TB control actions, problems in the organization of the material for bacteriological examination, inadequate approach to the patient during sputum collection and inadequate professional training [30]. It also highlights potential issues in TB transmission control, since “registration and examination of contacts” and “contacts examination for case finding” were included in this category. Taken together these findings suggest that this area requires priority attention from managers. This seems to be an international problem, since a meta-analysis published in 2013 identified a lack of systematization in the monitoring and control of the communicants, emphasizing that they represent a greater risk of exposure than the general population [31]. Evaluation of contacts was therefore considered a priority activity to reduce TB incidence of the disease, especially through chemoprophylaxis [31].

The professionals working in site C presented a significantly less favorable opinion in comparison to sites A and B, taken together. One possible explanation is the nature of site C: a high complexity hospital, dealing with multiresistant tuberculosis cases requiring better structural and organizational conditions, which have not yet been achieved in Pernambuco (e.g. more resources on contact tracing and transportation to TB treatment).

One of the strong points of this study is the insights provided on the perspectives of PCT patients and professionals involved directly or indirectly in their care. These stakeholders provided supplementary perspectives, which are useful to signal potential opportunities for improvement. The main limitation lies on the study samples. While all PCT patients in sites A, B and C were invited to participate, the response rate obtained (36%) raises the issue of whether respondents’ perspectives are representative of all users in these sites. Concerning professionals, the sample is non-probabilistic and relatively small, albeit the fact that the population (i.e. total number of eligible professionals in the sites) at the time of data collection was itself small. Respondents were selected based on convenience, and not “cherry picked” for their favorable or unfavorable views, which would have biased results. Nonetheless, the issue of representativeness of their views emerges again. As in other studies, the generalization of findings beyond study sites is uncertain, mainly due to small samples.

Larger studies are necessary and they should maintain a multi-professional approach, as the management of TB patients involves a multi-professional team. Pharmacists form a crucial part of this team and can be involved in different stages in the value chain for TB control [29].

## 5. Conclusions

Our research highlighted potential opportunities for improvement in the sites surveyed. Findings can be used by epidemiological surveillance and primary care managers as a starting point for a more in-depth analysis on the use of resources, particularly in respect to organisational accessibility.

The issue of how can the PCT in Pernambuco improve its performance seems unexplored. Combining evidence and expert opinion may prove valuable in deriving actions to improve program performance and consequently, reducing the risks posed by tuberculosis.

## Figures and Tables

**Table 1:** Patients’ (Pts) opinion on the PCT by item, Recife-PE, Brazil (items based on [9,15,16]).

Category	Item Number	Score	Item	Mean Score (SD)
Organizational accessibility (OA)	Pts-01	5–1	Was there a lack of TB medication during treatment?	4.4 (1.2)
	Pts-02	5–1	Was the waiting time for a TB medical consultation above 60 min?	2.9 (1.5)
	Pts-03	1–5	Was there access to a medical consultation in less than 24 hours when needed?	2.5 (1.8)
	Pts-04	1–5	Did health professionals perform home visits related to TB treatment?	2.0 (1.5)
	Pts-05	1–5	Did health professionals perform home visits for other reasons than TB?	1.9 (1.4)
	Pts-06	1–5	Was information on TB and its treatment provided?	2.7 (1.9)
	Pts-07	1–5	Was information on other health topics provided?	2.1 (1.7)
	Pts-08	1–5	Was there participation in groups of TB patients at the health unit?	1.1 (0.6)
	Pts-09	1–5	Was information about TB and its treatment provided to people living with the person with TB?	2.2 (1.6)
	Pts-10	1–5	Was advertising, campaigns or educational work performed by health service professionals?	1.6 (1.3)
	Pts-11	1–5	Did health professionals develop community actions for delivery of the sputum pot?	1.5 (1.15)
Economic accessibility (EA)	Pts-12	5–1	Were work or appointments missed to attend a TB medical consultation?	4.3 (1.4)
	Pts-13	5–1	Was it necessary to pay for transportation to attend a TB medical consultation?	3.5 (2.0)
	Pts-14	1–5	Did health professionals involve community leaders to discuss TB?	1.6 (1.3)
Geographical accessibility (GA)	Pts-15	5–1	Was it necessary to use motorized transport to attend a TB medical consultation?	2.5 (1.9)
	Pts-16	1–5	Was the nearest health unit attended for consultation?	3.0 (1.9)

**Table 2:** Professionals (Prs) opinion on the PCT by item, Recife-PE, Brazil (items based on 17).

Categories	Item Number	Score	Evaluation Criteria	Mean Score (SD)
Coverage and Reception (q1)	Prs-01	1–5	Professional attention	3.6 (0.9)
	Prs-02	1–5	Integrated care	3.3 (1.0)
Diagnosis, Clinical and Laboratory Assistance (q2)	Prs-03	1–5	Registration and examination of contacts	1.8 (0.9)
	Prs-04	1–5	Contacts examination for case finding	1.8 (1.0)
	Prs-05	1–5	Provision of rapid results for Smear Test	2.3 (0.7)
	Prs-06	1–5	Performing a sputum culture and sensitivity test when indicated	2.3 (0.8)
	Prs-07	1–5	Provision of rapid HIV test for TB patients	2.4 (0.6)
Treatment and Pharmaceutical Assistance (q3)	Prs-08	1–5	Availability and accessibility to treatment	4.3 (0.9)
	Prs-09	1–5	Referral of patients with multidrug-resistant TB to the referral center when indicated	4.2 (0.9)
	Prs-10	1–5	Supervised treatment for smear positive patients with risk of neglect and coinfection	3.1 (1.6)
Integrality of care (q4)	Prs-11	1–5	Provision of comprehensive care for all TB-related or non-TB-related health problems	3.0 (1.3)
	Prs-12	1–5	Provision of comprehensive prevention guidance for health promotion	3.1 (1.2)
